# The WD Domain of *Atg16l1* Crucial for LC3-Associated Phagocytosis Is Not Required for Preserving Skin Barrier Function in Mice

**DOI:** 10.1016/j.xjidi.2024.100283

**Published:** 2024-04-28

**Authors:** Shannon Conway, Matthew Jefferson, Derek T. Warren, Thomas Wileman, Christopher J. Morris

**Affiliations:** 1 School of Pharmacy, University of East Anglia, Norwich, United Kingdom; 2 Biomedical Research Centre, University of East Anglia, Norwich, United Kingdom; 3 Norwich Medical School, University of East Anglia, Norwich, United Kingdom; 4 Quadram Institute Bioscience, Norwich, United Kingdom; 5 UCL School of Pharmacy, London, United Kingdom

**Keywords:** Autophagy, Barrier function, Epidermal structure

## Abstract

The skin is a multifunctional organ, forming a barrier between the external and internal environment, thereby functioning as a safeguard against extrinsic factors. Autophagy has been implicated in epidermal differentiation and in preserving skin homeostasis. LC3-associated phagocytosis (LAP) uses some but not all components of autophagy. The *Atg16l1* (Δ WD) mouse model lacks the WD40 domain required for LAP and has been widely used to study the effects of LAP deficiency and autophagy on tissue homeostasis and response to infection.

In this study, the Δ WD model was used to study the relationship between LAP and skin homeostasis by determining whether LAP-deficient mice display a cutaneous phenotype. Skin histology of wild-type and Δ WD mice aged 1 year revealed minor morphological differences in the tail skin dermal layer. RT-qPCR and western blot analysis showed no differences in key keratin expression between genotypes. Skin barrier formation, assessed by dye permeation assays, demonstrated full and proper formation of the skin barrier at embryonic day 18.5 in both genotypes. Biomechanical analysis of the skin showed decreased skin elasticity in aged Δ WD but not wild-type mice. In summary, the LAP-deficient Δ WD mice displayed subtle alterations in dermal histology and age-related biomechanical changes.

## Introduction

The skin undergoes constant dynamic renewal, where a variety of stem and progenitor cells are activated in its 3 unique layers: the epidermis, dermis, and hypodermis (Joost et al, 2020). Keratinocytes lost from the superficial epidermis through desquamation or injury are replenished by the terminal differentiation of cells originating from a stem cell compartment found at the dermal–epidermal junction (Joost et al, 2020). In recent years, a role for autophagy has been implicated during epidermal differentiation and in maintaining skin homeostasis (Akinduro et al, 2016; Rossiter et al, 2013). Autophagy plays a key role in maintaining tissue homeostasis by degrading damaged proteins and organelles in lysosomes (Rai et al, 2019). Protein kinase B (Akt) and mTORC1, which inhibit autophagy, have been shown to be key regulators for epidermal development and differentiation and have been linked to conditions such as psoriasis (Akinduro et al, 2016). A pathway related to autophagy called LC3-associated phagocytosis (LAP) is activated in response to recognition of phagocytic cargoes by Toll-like receptors (Rai et al, 2019). LAP uses a subset of autophagy proteins to attach LC3/ATG8 to the cytosolic side of the phagosome membrane to facilitate fusion with lysosomes, thereby degrading material entering cells (eg, pathogens and protein aggregates) (Heckmann and Green, 2019). A LAP-deficient mouse model, known as Atg16l1^ΔWD^, lacks the WD domain of *Atg16l1*, which is required for LAP but not for autophagy (Rai et al, 2019). Atg16l1^ΔWD^ mice have been used to determine the roles played by autophagy and LAP in maintaining tissue homeostasis in vivo (Rai et al, 2019; Slowicka et al, 2019). LAP is reported to be critical in orchestrating mucosal defences to microbial infection, including influenza (Wang et al, 2021) and *Listeria monocytogenes* (Gluschko et al, 2022). The Atg16l1^ΔWD^ mouse model also develops spontaneous Alzheimer’s disease because the WD domain of *Atg16l1* is required for efficient clearance of β-amyloid and prevention of neuroinflammation (Heckmann et al, 2020). Collectively, these observations indicate a major role for the *Atg16l1* WD domain in barrier function and in reducing inflammation and led us to hypothesise that Atg16l1^ΔWD^ mice would display a cutaneous phenotype. In this study, we undertook histological and biochemical analyses of skin from wild-type (WT) and Atg16l1^ΔWD^ mice to identify phenotypic differences between the models. We identified differences in regional skin histology but, critically, no impairment of the skin barrier function.

## Results and Discussion

**Figure 1:**
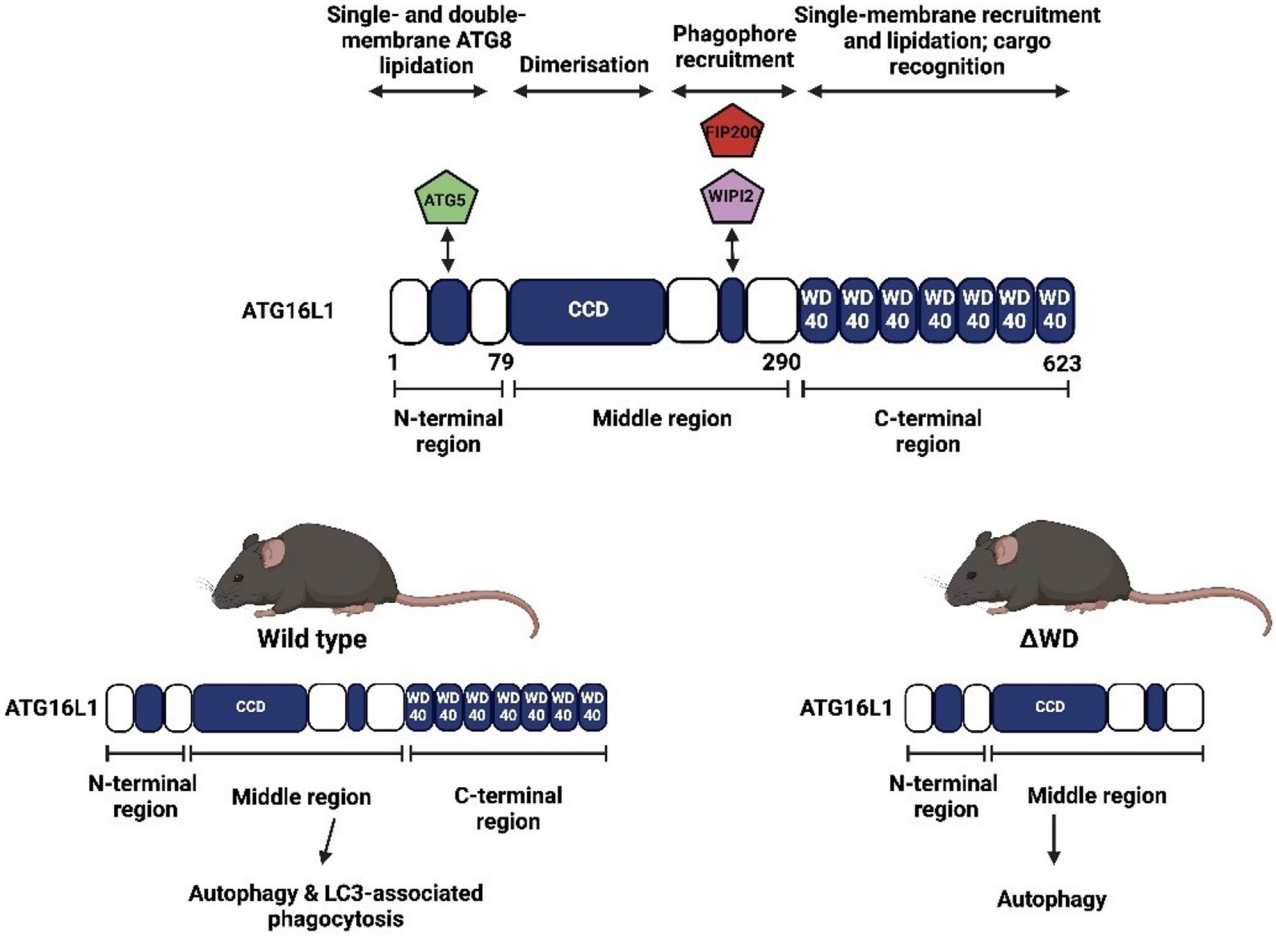
**Overview of Atg16l1^ΔWD^ mouse model.** Removal of the WD domain leading to LAP deficiency. Created with BioRender.com.

**Figure 2:**
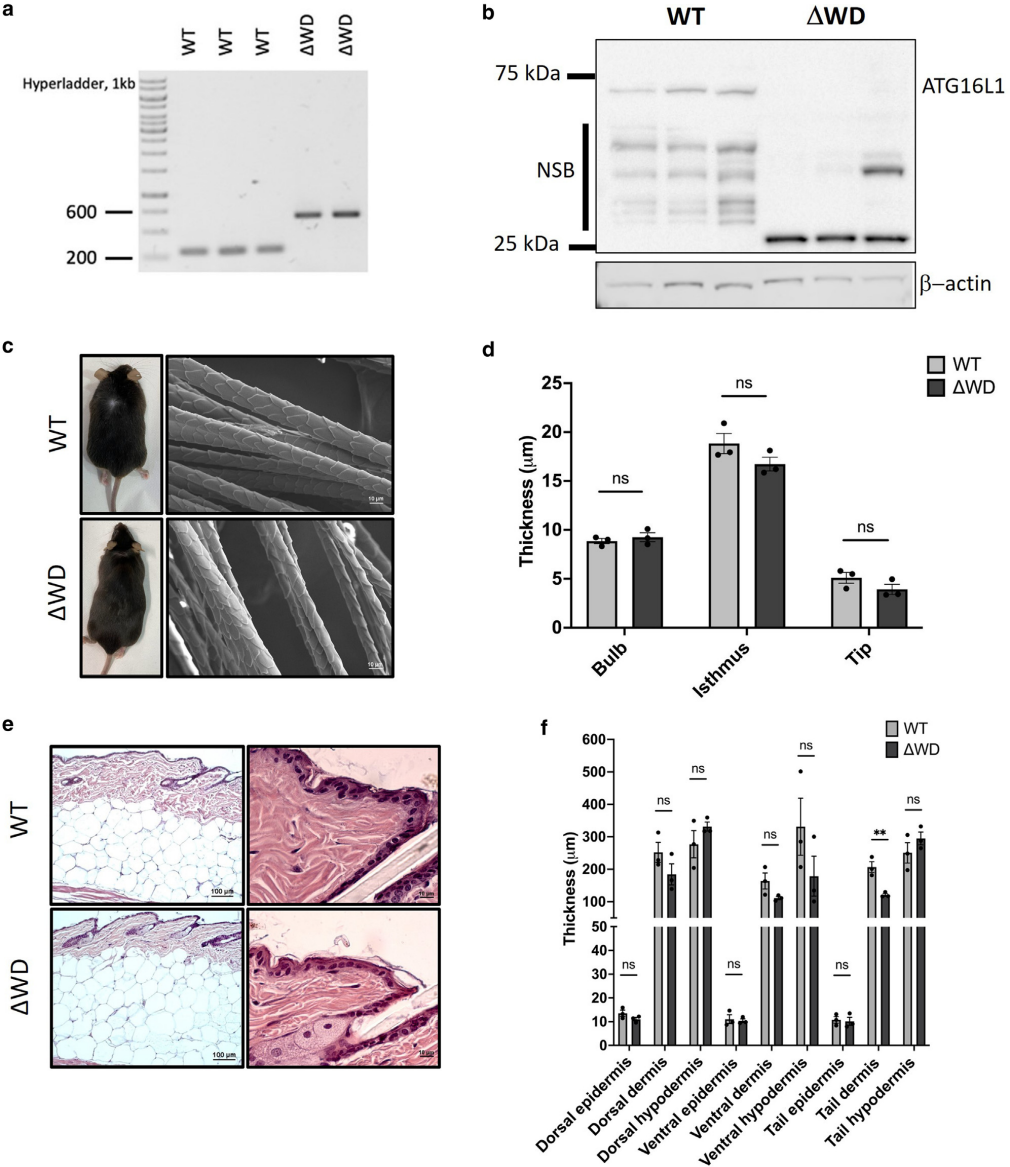
**Histological and morphological assessment of the integument.** (**a**) Exemplar 1% agarose gel showing genotyping for the Atg16l1^ΔWD^ mouse model. WT bands were expected at 291 bp, whereas ΔWD bands were predicted at 639 bp. (**b**) Upper: truncated ATG16L1 expression in the skin of ΔWD mice was confirmed by western blot, with the truncated form detected at approximately 25 kDa and the WT ATG16L1 detected at approximately 75 kDa. Representative blots are shown for n = 3 mice of each genotype. NSB denotes nonspecific antibody binding. Lower: β-actin loading control. (**c**) Left: overall appearance of WT and ΔWD mice. Right: scanning electron microscopy of hair plucked from WT and ΔWD dorsal skin. Bar = 10 μm. (**d**) Analysis of hair width at 3 distinct hair regions. Measurements taken at bulbar region, isthmus, and tip using ImageJ. Data shown are mean ± SE of n = 30 measurements taken per mouse (n = 3). Multiple unpaired *t*-test was performed. (**e**) Representative H&E histology of dorsal skin from WT and ΔWD mice. Low magnification image (left) and higher magnification image (right) bar = 100 μm (left) and 10 μm (right), respectively. (**f**) Quantification of skin layer thickness in dorsal, tail, and ventral skin. Individual means are shown; error bars are SE. n = 3 mice per genotype; n = 20 measurements per mouse. Multiple unpaired *t*-tests with Welch correction were used to compare group means at the various anatomical locations. ∗∗*P* < .01. ns, not significant; SE, standard error; WT, wild-type.

All experimental mice from the WT and Atg16l1^ΔWD^ (referred to as Δ WD in the remaining parts of this paper and described in Figure 1) groups were routinely genotyped (Figure 2a). We first sought to confirm that the truncated ATG16L1 protein was expressed in the skin. Western blotting of isolated epidermal tissue confirmed that WT mice expressed the full-length protein at approximately 75 kDa, whereas Δ WD littermates expressed the truncated protein, which was detectable at approximately 25 kDa (Figure 2b). Identical fur pigmentation (Figure 2c, left) was observed in both genotypes, with only a few white hairs present in both genotypes at age 1 year. The integument of WT and Δ WD littermates aged 1 year was further assessed by electron microscopy and Hematoxylin & Eosin (H&E) staining. Scanning electron microscopy analysis (Figure 2c, right) revealed no visual difference in hair morphology, and quantitative analysis revealed identical dimensions in both groups (Figure 2d). The thickness of the individual skin strata was measured at 3 anatomical locations, with representative H&E images of dorsal skin shown in Figure 2e. No significant differences in skin layer thickness were noted between genotypes, except for the dermal layer, which tended to be thinner in ΔWD mice across the 3 regions but reached significance (*P* < .01) only in the tail region (Figure 2f). The depth of hypodermal/subcutaneous fat was consistent between groups, as expected from our previous observation (Rai et al, 2019) that ΔWD mice display the same body mass growth rate as WT littermates.

**Figure 3:**
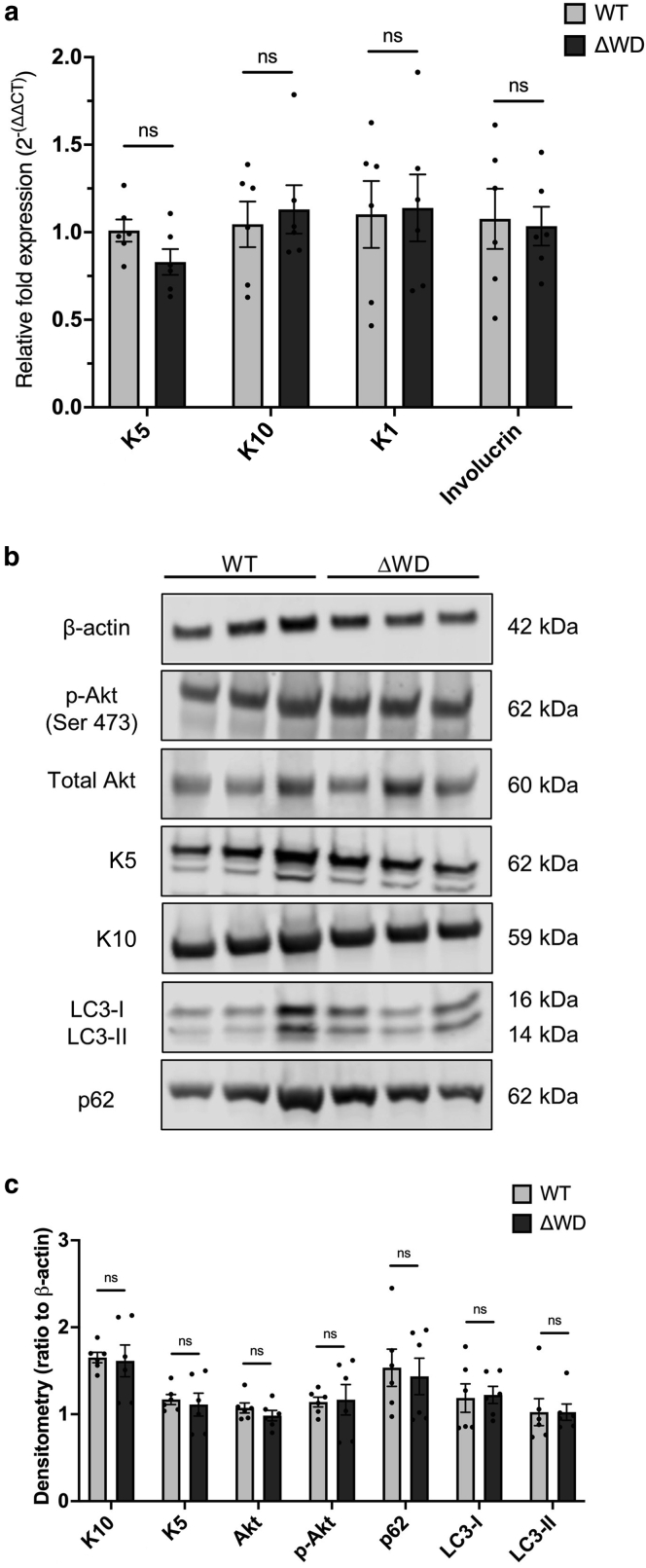
**Gene and protein expression in isolated epidermis tissue from WT and ΔWD mice** (**a**) RT-qPCR analysis of key keratins (K5, K10, and K1) and differentiation marker involucrin from isolated epidermal tissue. Relative mRNA expression was normalized to 18S. Individual data points are mean of triplicate measurements of cDNA samples from individual mice. Error bars are SE. n = 6 mice per genotype. Multiple unpaired *t*-tests were used. (**b**) Representative western blots for K5, K10, Akt, p-Akt, LC3-I/II, and p62 and β-actin. (**c**) Densitometric analysis of western blot data in panel **b**. Band densities were quantified and normalized to the β-actin loading control (n = 3). n = 6 mice per genotype. Multiple unpaired *t*-tests were performed. Error bars are SE. Akt, protein kinase B; K, keratin; ns, not significant; p-Akt, phosphorylated protein kinase B; SE, standard error; WT, wild-type.

Epidermal permeability and barrier function of the skin are closely linked to the precisely controlled turnover and replacement of epidermal cell layers. Indeed, dysregulation of such results in skin diseases, including psoriasis (epidermal thickening) and age-related skin thinning (Akinduro et al, 2016; Harn et al, 2021). RT-qPCR analysis revealed no change in the basal layer marker, keratin (K)5 gene *K5*, and the upper epidermal markers, *K1*, *K10*, and involucrin gene *IVL*, between WT and Δ WD mice (Figure 3a). Western blotting for K5 and K10 (Figure 3b and c) demonstrated that these key intermediate filament proteins are expressed equally in the epidermis of both models and thus corroborated the RNA expression data. The expression levels of the total Akt and the activated phosphorylated Akt proliferative marker were comparable (Figure 3c). This indicates that the WT and Δ WD mice display normal epidermal proliferation and differentiation. Absolute and relative expression levels of LC3-I and LC3-II in the epidermises (Figure 3b and c) were equal in both models, indicating that under steady-state conditions, there was no differential activation of LAP in the Δ WD model compared with that in the WT mice. These results are consistent with previous work in embryonic fibroblasts derived from these mice (Wang et al, 2021). Canonical autophagic flux can be examined through detection of p62 levels. Both models expressed similar levels of p62 (Figure 3b and c), which indicates that deletion of the WD domain of *Atg16l1* had no effect on the overall autophagic flux in the skin.

**Figure 4:**
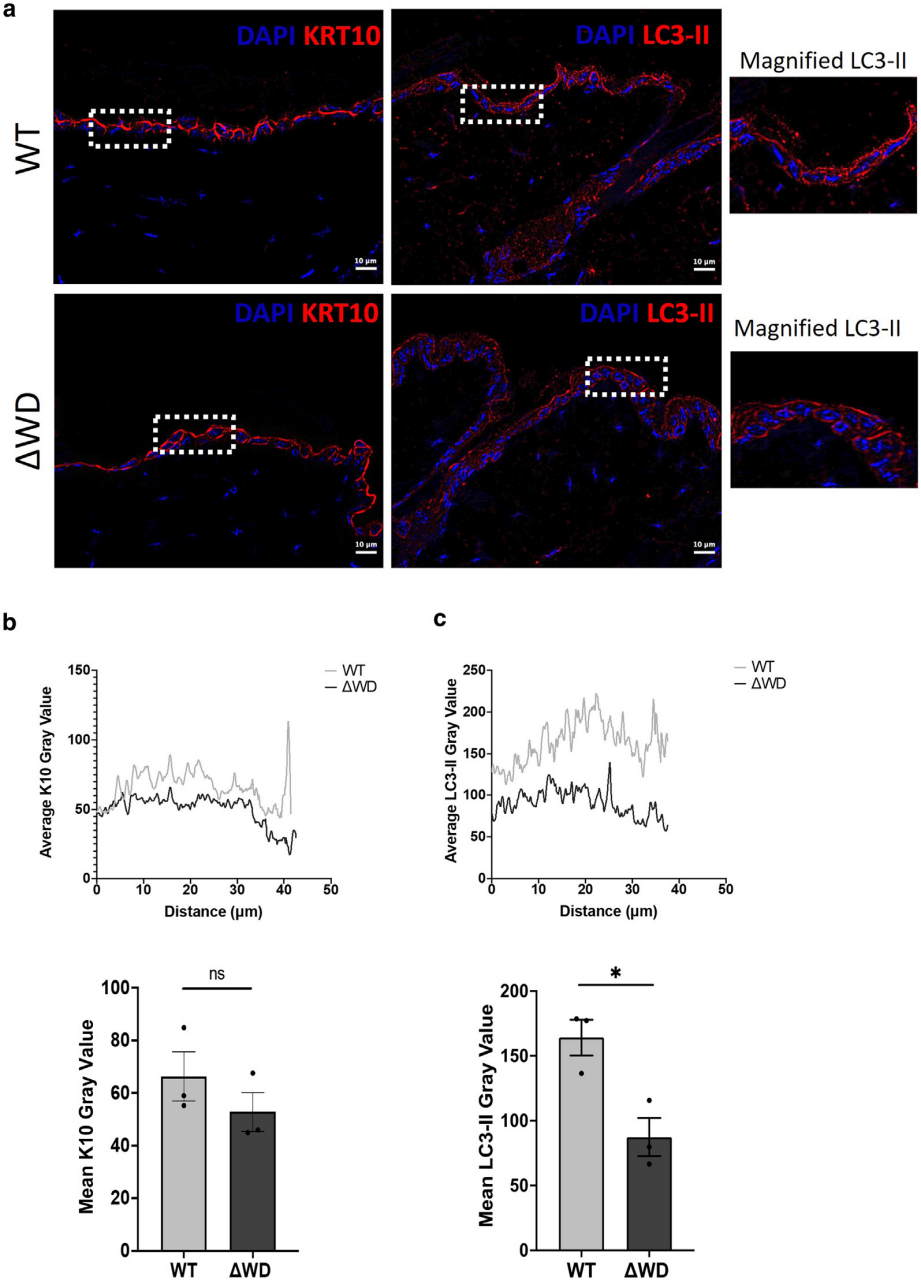
**LC3-II and K10 expression in mouse skin at 1 year.** (**a**) Representative immunohistochemistry of paraffin-embedded dorsal skin showing K10 (left) and LC3-II (right) in the ΔWD and WT littermate controls. Bar = 10 μm. Magnified images of the areas marked by a dashed box are shown on the right. Images are representative from 3 independent experiments on n = 3 mice per genotype. (**b, c)** Upper panels: fluorescence intensity profile plots showing K10 and LC3-II staining intensity in the adult WT and ΔWD skin quantified in Fiji ImageJ. Intensity profiles were measured across skin sections as indicated by the representative ROI shown in panel **a**. Data are derived from n = 3 measurements per mice from n = 3 mice per genotype. Lower panels: mean fluorescent intensities of the K10 and LC3-II gray value ± SE. n = 3 mice per genotype and n = 6 measurements per mouse. Means were compared using the unpaired *t*-test. ∗*P* < .05. K10, keratin 10; ns, not significant; ROI, region of interest; SE, standard error; WT, wild-type.

We further characterized the expression of LC3 using immunohistochemistry on full-thickness skin sections. We stained for the suprabasal epidermal marker K10 as an internal control protein and found that it was consistently localized above the basal layer in both genotypes (Figure 4a, left). Immunolocalization of LC3 across the follicular and intrafollicular epidermis of WT and Δ WD mice was undertaken using an antibody specific for the lipidated LC3 form (Figure 4a, right hand images and magnified inserts). In the skin of WT mice, the LC3-II staining appeared slightly more punctate in the cytoplasm with pronounced staining around the perinuclear region. In contrast, LC3-II staining in the Δ WD epidermis appeared slightly weaker and located at the cell periphery (Figure 4a, magnified inserts). Technical limitations prevented us from performing higher-resolution imaging of these tissue sections, but overall, this staining pattern was consistent with the spatial distribution of nonlipidated LC3 and the LC3–GFP fusion protein in adult mouse skin reported by others (Akinduro et al, 2016; Rossiter et al, 2013). Quantitative fluorescence intensity analysis of K10 across horizontal sections of the skin was consistent (*P* > .05) in both genotypes (Figure 4b). In contrast, the overall fluorescence levels of LC3-II were reduced (*P* < .05) in the Δ WD model (Figure 4c). Considered together with the western blot data in Figure 3b, we interpret this finding as a spatial redistribution of LC3-II within the epidermis in the mutant mouse. Under steady-state conditions, the truncation of ATG16L1 in the Δ WD model is not expected to affect the distribution of lipidated LC3 within the cell. Wang et al (2021) previously showed that in nonphagocytic skin fibroblasts, the Δ WD model prevents the recruitment of LC3-II to large vacuolar structures after treatment with chloroquine and monensin but was equivalent in control (Hanks' Balanced Salt Solution)-treated cells.

**Figure 5:**
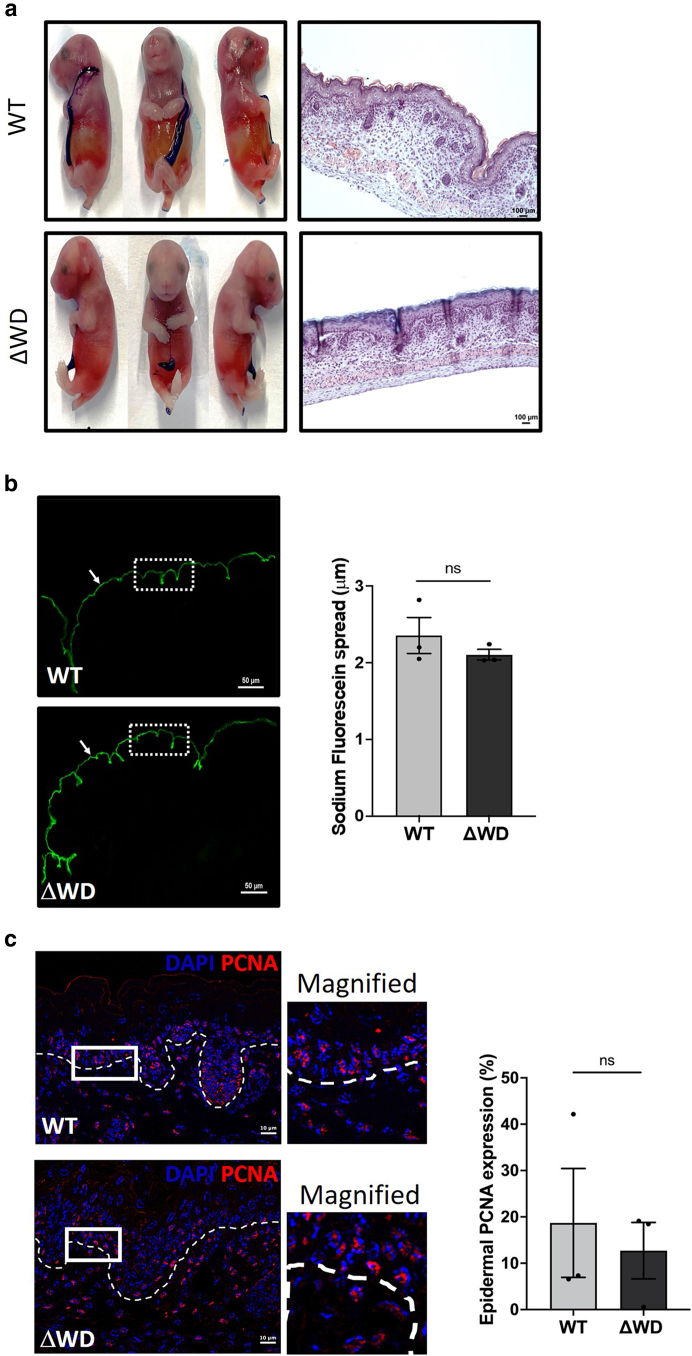
**Assessment of the Atg16l1^**ΔWD**^ mouse model skin barrier in embryos at E18.5 revealed an intact skin barrier.** (**a**) Left: dye permeation assay on embryos at E18.5 using 0.1% toluidine blue dye. Right: H&E staining of full-thickness embryonic skin. Bar = 100 μm (**b**) Left: epidermal permeability assay using sodium fluorescein dye. Bar = 50 μm. Images are representative from 3 independent experiments. Arrows highlight the continuous sodium fluorescein staining of the corneal epidermal layer. Right: quantification of sodium fluorescein permeation depth. Data shown are mean depth of sodium fluorescein permeation ± SE. n = 3 mice per group with n = 40 measurements per mouse. Mann–Whitney test was used for comparison. (**c**) Left: representative immunohistochemistry of nuclear protein PCNA in the embryonic skin. Bar = 10 μm. Magnified image of ROI box is shown to the right of the micrograph. Dashed line indicates the dermal–epidermal boundary. Right: quantification of epidermal PCNA expression. Data are mean (%) ± SE with n = 3 per group; n = 2 epidermal regions per mouse were counted using Fiji ImageJ. Mann–Whitney test was performed. E18.5, embryonic day 18.5; ns, not significant; PCNA, proliferating cell nuclear antigen; ROI, region of interest; SE, standard error; WT, wild type.

Intact skin permeability was assessed using dye permeation assays on embryos at embryonic day 18.5. At this stage of development, the epidermal strata have fully developed, and the absence of the hair follicles presents an uninterrupted epidermal surface barrier (Figure 5a), the permeability of which can be assessed using toluidine blue and sodium fluorescein dyes. No changes in the extent of toluidine blue retention in the skin were noted (Figure 5a); dye retention was noted only at the umbilicus and sites of tail cutting for genotyping. To gain a deeper insight into the skin permeability, we analyzed the depth of sodium fluorescein dye penetration into the embryonic skin using fluorescence microscopy of skin sections (Figure 5b, left). In both models, the skin presented a homogenous and continuous band of sodium fluorescein staining, as indicated by the arrow in Figure 5b, measurements of which showed a consistent depth (*P* > .05) of approximately 2 μm in both WT and Δ WD (Figure 5b, right). The number of replicative cells in the embryonic skin was determined by measuring the proportion of proliferating cell nuclear antigen–positive cell nuclei (Figure 5c). There was no significant difference between WT and Δ WD skin, as would be expected from the thickness of both adult (Figure 2e and f) and embryonic (Figure 5a and b) skin. This indicates that the loss of LAP functionality has no detrimental effect on the barrier function of the epidermis. This finding is consistent with a previous study that showed that a skin-targeted deletion of the key autophagy protein, ATG7, in mice did not produce an epidermal phenotype, notwithstanding dramatic effects on canonical autophagy (Rossiter et al, 2013).

**Figure 6:**
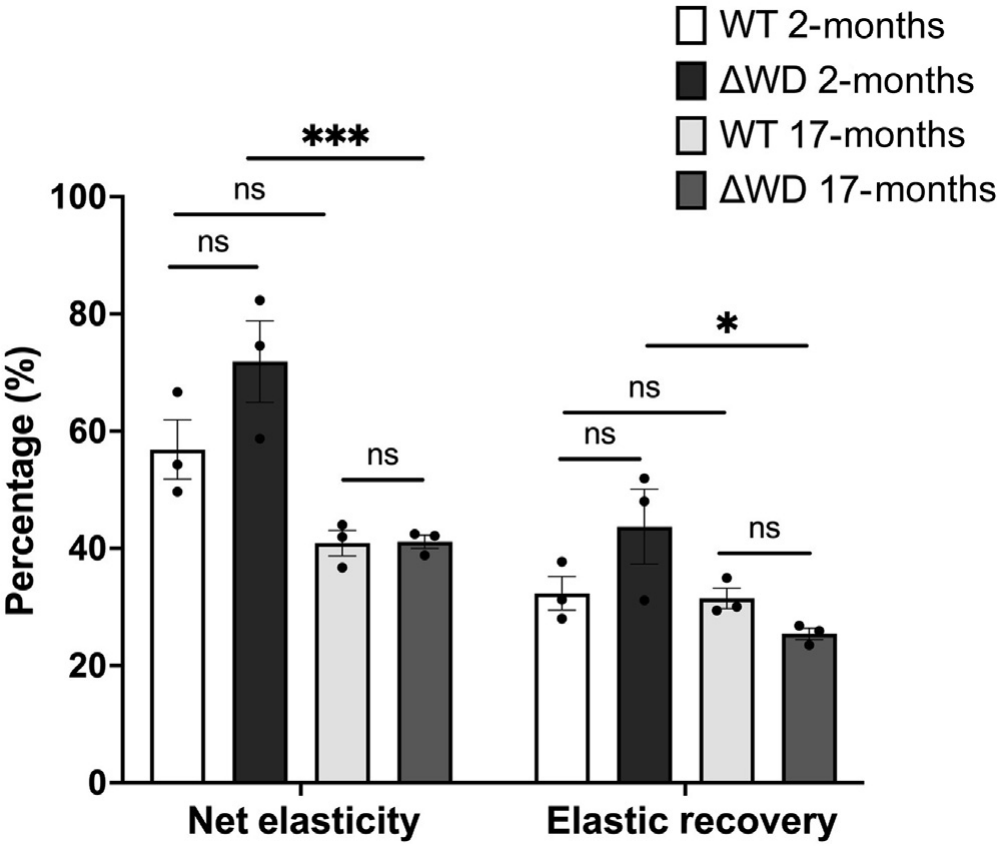
**Net elasticity and overall elastic recovery in WT and ΔWD mouse skin.** Net elasticity and elastic recovery were measured using the Cutometer MPA580. Net elasticity highlighting that aged (17 months) ΔWD mice exhibit decreased net elasticity and elastic recovery compared with those at 2 months. Data represent the mean of n = 6 measurements taken per mouse. Error bars are SE. n = 3 mice per genotype. Two-way ANOVA with Tukey’s posthoc test was performed. ∗ *P* < .05 and ∗∗∗ *P* < .001. ns, not significant; SE, standard error; WT, wild type.

As is the case for humans, the mechanical and structural properties of murine skin change with age (Chen et al, 2017; Harn et al, 2021). The dermal layer provides mechanical and structural support to the skin and serves as a cushion for the blood and lymphatic perfusion circuits. To complete our cutaneous examination of this model, we questioned whether the gross biomechanical functionality may be compromised in the Δ WD mouse. To test this, we evaluated the elastic function of the mouse skin at ages of 2 and 17 months. The net elasticity refers to the ability of the skin to return promptly to its relaxed state after deformation, whereas the elastic recovery parameter denotes the relative speed of recovery as a proportion of the total deformation (Abbas et al, 2022). The net elasticity and elastic recovery of both genotypes were statistically indistinguishable at the matched ages of 2 and 17 months (Figure 6). In ΔWD mice, both elasticity parameters decreased with age. In the WT mice, the net elasticity showed a decreasing trend but did not reach statistical significance. The elastic recovery of WT skin was similar at both ages. This indicates that the loss of LAP functionality over a lifetime of 17 months accelerates intrinsic skin aging relative to WT. Further studies on more extensively aged mice and with higher statistical power would offer deeper insights into the cutaneous phenotype of the ΔWD mice. Age-related physiological/pathophysiological changes in other tissues might be different in WT and Δ WD mice. For example, Δ WD mice aged 2 years displayed a greater abundance of β amyloid and hyperphosphorylated tau and neuroinflammation in the brain than WT littermates, which was associated with a progressive neurodegeneration and memory impairment consistent with spontaneous Alzheimer’s disease (Heckmann et al, 2020). In *Caenorhabditis elegans,* the *Atg16l1* orthologue, *atg-16.2*, and the WD40 domain in particular are key components of the noncanonical autophagy processes that may be critical to tissue maintenance and healthy organismal lifespan (Yang et al, 2024).

In conclusion, the histological examination of male Δ WD mouse skin at age 1 year revealed a modest and anatomically restricted skin histology phenotype in healthy skin compared with that of WT. The differences were shown to be functionally insignificant in terms of epidermal permeability but caused an age-related deterioration in dermal elasticity. The cutaneous response to cellular offences (eg, viral or bacterial infection) in the Δ WD mice may exhibit defective responses (eg, cytokine release), which remains an ongoing study in our laboratory.

## Materials and Methods

### Mice

The generation of the Atg16l1^ΔWD^ mice has been previously described (Rai et al, 2019) and is summarized in Figure 1. Generation and breeding of mice for the genotype was approved by the University of East Anglia Animal Welfare and Ethical Review Body and performed under UK Home Office Project License 70/8232. Every mouse used in each experiment was genotyped using PCR primers described previously (Rai et al, 2019).

### Whole-skin isolation and immunohistochemistry

Dissected mouse skin was placed in a histology cassette and fixed in 10% neutral buffered formalin (Sigma-Aldrich) overnight at room temperature. The following day, the tissue was washed, dehydrated through a graded series of ethanol solutions, and paraffin wax embedded. This process enables the generation of a solid cuboidal block containing the skin tissue, which can be easily mounted onto a microtome (Microm, HM355S) and stored at 4 °C for long-term storage. Tissue sections (5 μm) were cut using specific microtome blades (MX35 Ultra, Thermo Fisher Scientific) and were carefully transferred onto coated glass slides (Thermo Fisher Scientific, Superfrost Plus) and left to dry overnight at room temperature. Slides were labeled and stored at 4 °C until further processing.

### H&E staining of whole-skin tissue

Tissue sections were H&E stained by submerging the slides in Mayers hematoxylin (Thermo Fisher Scientific) and Eosin Y (Thermo Fisher Scientific) solutions. Once the sections have passed through each solution, they were then mounted using coverslips (VWR International) and mounting medium (DPX, Thermo Fisher Scientific). Sections were examined under the brightfield Apotome 3 Imager.Z2 microscope. Brightfield images of H&E sections were analyzed using the straight-line measurement tool in ImageJ Fiji (1.48v) (Schindelin et al, 2012). A total of 20 measurements were taken in each distinctive skin layer at various anatomical locations (ie, dorsal, ventral, and tail) across 3 WT and 3 ΔWD mice. Multiple unpaired *t*-tests with Welch correction were used to compare both genotypes at various anatomical locations.

### Immunolabeling of skin tissue

Sections of 5-μm thickness were cut from each paraffin block and were placed on charged slides (Superfrost Plus slides, Thermo Fisher Scientific) and dried overnight at room temperature. Sections were dewaxed and rehydrated before heat-mediated antigen retrieval using 0.1 M citrate buffer at pH 6.0 for 10 minutes at 95 °C. Tissue was permeabilized using 0.5% Tween-20 solution for 15 minutes. Endogenous peroxidase blocking was performed using 3% hydrogen peroxidase in 100% methanol for 20 minutes. Nonspecific background blocking was performed using 10% normal goat serum in PBS at room temperature for 30 minutes. Primary antibodies were diluted in 10% normal goat serum in PBS at their desired concentrations and were incubated at 4 °C overnight in a humidified chamber. Rabbit LC3B (LC3-II) (1:500) (Sigma-Aldrich, L8918), goat anti-rabbit Alexa Fluor 546 (1:1000) (Thermo Fisher Scientific, A-11035), rabbit antiproliferating cell nuclear antigen (1:200) (Proteintech, 10205-2-AP) and anti-cytokeratin 10 (1:500) (Abcam 76318) were used. Tissue sections were mounted with Vectashield with DAPI (Vector Laboratories). Slides were imaged using the Apotome 3 Imager.Z2. Fluorescence intensity was quantified using ImageJ Fiji (1.48v) (Schindelin et al, 2012). The unpaired *t*-test was used to compare fluorescent intensities of LC3-II and K10 in both genotypes.

### Scanning electron microscopy

Dorsal hair was plucked from the skin and mounted on aluminium stubs covered with conductive carbon tape and then gold coated with a Polaron SC7640 high-resolution sputter coater, manufactured by Quorum Technologies. The hair samples were imaged under vacuum with the Zeiss Gemini 300 scanning electron microscope using the secondary electron detector. Analysis was conducted using ImageJ Fiji (1.48v) (Schindelin et al, 2012). Multiple unpaired *t*-tests were used to compare the means of hair morphology in both genotypes.

### Epidermis tissue harvest

The entire mouse tail was removed and placed in PBS/Betadine solution (1:1), followed by a wash in PBS and rinse in 70% ethanol. Once the tail was washed, it was stored in a kanamycin solution (50 μg/ml in PBS) until further processing. Skin was removed from the tail bone and placed epidermal side down in a sterile petri dish. The hypodermal layer was carefully removed before it was placed into a new sterile petri dish. The tail skin was incubated dermal side down in ice-cold 0.25% trypsin without EDTA (speciality media) for 2 hours at 37 °C. After incubation, the epidermis was peeled off with curved tweezers and stored accordingly (ie, in RNA later for mRNA analysis or snap frozen for protein extraction) before further processing.

### Western blot

Frozen tail skin was homogenized in a pestle and mortar and lysed in urea buffer (6.5 M urea, 1 mM dithiothreitol, 50 mM Tris-hydrogen chloride). Skin lysate (1.5 μl) was denatured at 95 °C in SDS sample loading buffer and loaded into Bolt 4–12% Bis-Tris SDS-PAGE gels (Invitrogen). The gels were electrophoresed alongside the Precision Plus Dual Colour protein standards (Bio-Rad Laboratories) in NuPAGE MES SDS (Invitrogen) running buffer for 30 minutes at a constant voltage of 200 V. Proteins were transferred onto polyvinylidene fluoride membrane (Sigma-Aldrich) using the Bio-Rad Trans-Blot cell at constant 200 mA for 1 hour. Nonphosphorylated protein detection was carried out on polyvinylidene fluoride membranes that were blocked in 5% skimmed milk in Tris-buffered saline plus 0.1% (v/v) Tween 20. Phosphorylated proteins were detected after membrane blocking in 5% BSA in PBS. The following primary and secondary antibodies were used in the analysis: anticytokeratin 10 (1:10,000, Abcam, 76318), cytokeratin 5 (1:1000, Sigma-Aldrich, SAB4501651), LC3A/B (1:1000, Cell Signaling Technology, 4108S), anti-ATG16L (1:1000, MBL International, M150-3), anti-SQSTM1/p62 (1:1000, Cell Signaling Technology, 5114S), Akt (pan) (1:1000, Cell Signaling Technology, 9272S), phosphorylated Akt (1:1000, Proteintech, 66444-1-Ig), β-actin (1:10000, Sigma-Aldrich, A1978), IRDye 800CW (1:10,000, Li-Cor, 926-32210), and IRDye 680RD (1:10,000, Li-Cor, 926-68071). Blots were imaged using the Odyssey CLX imaging system (Li-Cor). Densitometry was performed using ImageJ Fiji (1.48v) (Schindelin et al, 2012). Multiple unpaired *t*-tests were used to compare protein expression in both genotypes.

### RNA extraction and RT-qPCR

Mouse tissue was removed from RNA later (Thermo Fisher Scientific) and cut into small pieces (approximately 2 mm) and transferred to a 2 ml Eppendorf safe-lock tube (Thermo Fisher Scientific) containing 600 μl TRIzol (Invitrogen) and one Tungsten Carbide bead (Qiagen). The tubes were placed in a tissue lyser (Qiagen, 85300) and were shaken twice for 5 minutes at 30 Hz. The homogenate was later centrifuged at 14,000*g* for 10 minutes at 4 °C. The supernatant was removed and added to a fresh centrifuge tube (Eppendorf) containing 200 μl of chloroform (Thermo Fisher Scientific). The tube was vortexed for 15 seconds and incubated at room temperature for 3–5 minutes. After incubation, the solution was centrifuged at 14,000*g* for 15 minutes at 4 °C to separate the phases. The upper aqueous layer was removed and added to a fresh centrifuge tube containing 200 μl of 95% ethanol. The solution was mixed before proceeding with total RNA extraction using the GenElute total RNA purification kit (Sigma-Aldrich, RNB100), following manufacturer’s instructions. RNA was quality controlled using the Nanodrop 2000 (Thermo Fisher Scientific).

RNA was reverse transcribed to cDNA using random hexamers (50 μM), 10 mM 2′-deoxynucleoside 5′-triphosphate (New England Biolabs), 5× first-strand buffer (Invitrogen), 0.1 M dithiothreitol (Invitrogen), SuperScript II RT (Invitrogen), and 1 ng–5 μg total RNA. Predesigned KiCqStart SYBR Green primers were purchased from Sigma-Aldrich (Table 1). SYBR Green qPCR was performed using the QuantStudio 3 Real-Time PCR System (Thermo Fisher Scientific) using MicroAmp optical 96-well reaction plates (Applied Biosystems). Relative quantification was calculated using the 2ˆ (-ΔΔ)CT method using *18S* as a reference gene. Multiple unpaired *t*-tests were used to compare the RNA expression data across genotypes.

**Table 1:** qPCR Primers

Gene	Context Length Sequence (bp)	Sequence (5′–3′)
*18S*	22 21	Forward: GCCGCTAGAGGTGAAATTCTTG; reverse: CATTCTTGGCAAATGCTTTCG
*K5*	21 20	Forward: GTGATGTTGAAGAAGGATGTG; reverse: TTCATGAAGTTGATCTCGTC
*K10*	20 21	Forward: CAATCAGAAGAGCAAGGAAC; reverse: CAGTGATTTCAGACTTATGGC
*K1*	22 20	Forward: CTACCAAATGGAAATGTCTCAG; reverse: GTAAAAGGTCTCAGCTTCAG
*IVL*	21 20	Forward: CTGTGAGTTTGTTTGGTCTAC; reverse: GAAAGCCCTTCTCTTGAATC

Abbreviations: K, keratin; IVL, involucrin.

### Timed mating and embryo isolation

For the timed pregnancy, the triomating method (1 male with 2 females per genotype) was used. Matings were set up the evening before the start of the dark cycle. Females were separated from the male mouse upon confirmation of a vaginal plug the following day. This is considered as gestation day 0.5 (embryonic day 0.5). If no plug was observed, the female mouse was rehoused with the same sire for a maximum of 5 days. Embryos were harvested and killed by anaesthetic overdose at embryonic day 18.5—1 day before birth—to ensure the full development of the skin barrier without the presence of hair follicles.

### Skin barrier permeability studies

Toluidine blue assay was performed on unfixed, whole-mouse embryos (embryonic day 18.5). The embryos were isolated from the pregnant female at embryonic day 18.5 that had been killed by anaesthetic overdose (Euthatal, pentobarbital sodium, 200 mg in 1 ml). Embryos were immersed in ice-cold PBS for 30 minutes. The embryos were passed through a chilled methanol gradient (2 minutes per step). After passing the embryos through methanol, they were then immersed in 0.1% toluidine blue (Sigma-Aldrich) solution in water for 1–2 minutes on ice. The embryos were destained in PBS (pH 7.4) until a dye pattern was visible.

A sodium fluorescein permeation assay was performed on unfixed, whole-mouse embryo (embryonic day 18.5) dorsal skin. Embryos were placed epidermal side down in a petri dish containing 1 mM sodium fluorescein in PBS (pH 7.4) at 37 °C for 1 hour. After 1-hour incubation, the dorsal skin was removed and fixed in 10% neutral buffered formalin (Sigma-Aldrich). After fixation, the tissue was dehydrated and processed as described earlier. Slides were rehydrated, mounted, and imaged with the Zeiss Apotome 3 Imager.Z2. Fluorescent dye penetration was quantified using ImageJ Fiji (1.48v) (Schindelin et al, 2012). The Mann–Whitney test was used to assess sodium fluorescein dye permeation.

### Skin elasticity measurements

The Cutometer MPA580 (EnviroDerm) with a 2-mm diameter probe was used in the assessment of skin elasticity of WT and ΔWD mice immediately post-mortem after killing at 2 or 17 months. The pressure in the probe was set to 400 mBar with 5 seconds of suction, followed by 5 seconds of release. To examine the elasticity in further detail, the following parameters were considered: net elasticity (Ur/Ue) and elastic recovery (Ur/Uf). Parameters were calculated in the MPA CTplus software. Further detail can be found in Table 2. A 2-way ANOVA with Tukey’s posthoc was used to compare the means among the various groups.

**Table 2:** Overview of the Cutometer Parameters

Parameter	Definition
Uf	Total distance the skin stretches after the suction period
Ue	Distance the skin stretches in the initial 0.1 seconds of suction
Ur	Distance the skin retracts in the initial 0.1 seconds of the relaxation phase
Ur/Ue	Elastic part of the suction phase versus immediate recovery during the relaxation phase
Ur/Uf	Proportion of the immediate recovery compared with the amplitude after suction (as a percentage)

### Statistical analysis

Continuous data are presented as the mean ± SEM. For comparisons between groups, multiple unpaired *t*-tests with the 2-stage step-up (Benjamini, Krieger, and Yekutieli method), unpaired *t*-tests, or Mann–Whitney *U* tests were used, whereas comparisons among multiple groups used a 2-way ANOVA with Tukey’s posthoc test. Prior to testing, Levene’s test was used to evaluate the homogeneity of the variances, and a Shapiro–Wilk test was used to determine data normality. A *P* < .05 was considered statistically significant, and analyses were performed in GraphPad Prism, version 10.2.0, for Macintosh (GraphPad Software, Boston, MA) or SPSS, version 29.0.1.0 (released 2022, IBM SPSS Statistics for Macintosh, version 28.0, IBM, Armonk, NY).

## Ethics Statement

All experiments were performed in accordance with UK Home Office guidelines and under the UK Animals (Scientific Procedures) Act 1986.

## Data Availability Statement

No datasets were generated or analyzed during this study. Data related to this article will be made available by the authors without undue reservation.

## ORCIDs

Shannon Conway: http://orcid.org/0009-0001-4306-8983

Matthew Jefferson: http://orcid.org/0000-0003-2133-9487

Derek T. Warren: http://orcid.org/0000-0003-0346-7450

Thomas Wileman: http://orcid.org/0000-0002-9033-2580

Christopher J. Morris: http://orcid.org/0000-0002-7703-4474

## Conflict of Interest

The authors state no conflict of interest.
